# Prevalence of sexual activity and associated factors in hypertensive males and females in China: A cross-sectional study

**DOI:** 10.1186/1471-2458-12-364

**Published:** 2012-07-12

**Authors:** Xiaojun Chen, Qingying Zhang, Xuerui Tan

**Affiliations:** 1 Department of Cardiology, The First Affiliated Hospital of Shantou University Medical College, Shantou, Guangdong 515041, China; 2 Department of Preventive Medicine, Shantou University Medical College, Shantou, Guangdong 515041, China

**Keywords:** Sexual activity, Hypertensive, Cross-sectional survey, Chinese population

## Abstract

**Background:**

Hypertension is an important factor contributing to sexual dysfunction. The number of people with hypertension is increasing in China, but research into sexual life, which has implications for quality of life, is limited. We aimed to compare sexual activity and the influence of daily behaviors and sexual domain of hypertensive males and females in south China.

**Methods:**

A cross-sectional study was conducted at the health care center of a university-affiliated hospital from 2007 to 2008. We enrolled 502 subjects with hypertension (225 males, 48.79 ± 7.39 years old; 277 females, 48.26 ± 6.93 years old) and 173 with normotension (82 males, 45.69 ± 6.58 years old; 91 females, 46.14 ± 7.03 years old), all sexually active. All subjects completed a self-administered questionnaire on sexual activity before a routine physical check-up. Data were collected on sociodemographic and clinical characteristics, use of cigarettes and intake of beverages (including alcohol).

**Results:**

Hypertensive and normotensive subjects differed in frequency of orgasms and of sexual satisfaction, as well as duration of sexual activity. For hypertensive men, low frequency of sexual activity, orgasms and satisfaction were associated with unemployed or retired status than physical labor work (odds ratio [OR] 0.28 [95% confidence interval (95% CI) 0.12–0.69], 0.32 [0.12–0.86], 0.33 [0.19–0.88], respectively; *p* < 0.05), and long sexual duration was associated with never drinking alcohol than heavy drinking (OR 4.49 [1.28–6.41]). For hypertensive women, low frequency and duration of sexual activity and low satisfaction were associated with never drinking tea than heavy tea drinking (OR 0.42 [0.18–0.96], 0.49 [0.24–0.98], 0.29 [0.14–0.64], respectively; *p* < 0.05). Medication use and electrocardiography results were not associated with sexual activity for hypertensive patients.

**Conclusions:**

For hypertensive people in China, lifestyle factors are associated with sexual dysfunction, which differs by the sex of the person. Further research needs to examine serum hormone levels to validate the result.

## Background

Sexual problems are a common medical disorder related to illness, psychological status and social lives of the general population [1]. Anxiety and depression are psychological problems implicated in sexual dysfunction (SD), whereas marital status and relationships are socially related factors [2]. Chronic diseases such as diabetes mellitus and hypertension are common risk factors for SD in men [3-5]. Hypertension may contribute to SD, and many antihypertensive drugs might worsen sexual function because of side effects and decrease patient adherence to anti-hypertensive treatment [6]. Essential hypertension with its chronically evaluated blood pressure is considered a risk factor of cardiovascular disease, which is significantly associated with erectile dysfunction [7]. Erectile dysfunction is more frequent in patients with essential hypertension than normotensive subjects [8-10]. The prevalence of erectile dysfunction ranges from 15% in Brazil to 54% in Morocco [11]. The age-adjusted prevalence for Asians is 35% in Japan, 36.7% in Hong Kong and 28.3% in mainland China [12-14]. However, the large variation in prevalence may due to different sample populations, assessment methods and cultures.

Although male sexuality has been studied extensively, female SD in hypertension is less explored, and results vary because of small sample sizes. A study of 216 subjects in Greece found 42.1% of hypertensive women with SD, and the prevalence decreased with adequate blood pressure control [15]. Another study of 71 women found that most (90%) with essential hypertensive had SD [16]. In another study, hypertensive women showed lower vaginal lubrication, less frequent orgasms, and more frequent pain than normotensive women [17].

Because sexual activity may vary by culture and by race and ethnicity, the results from other countries may not apply to Chinese populations, whose sexual activity and risk factors are poorly understood. China is facing the critical challenge of the rapidly increasing prevalence of hypertension. The latest survey in a southern China area, Guangdong province, of 85 million residents found a 20.5% prevalence of hypertension [18]. Research into sexual function in western countries is abundant but limited in China. Thus, we aimed to investigate factors associated with the sexual domain and activity of people with hypertension in southern China.

## Methods

### Study design and population

This study involved a cross-sectional design. Data were collected from 2007 to 2008 in the First Affiliated Hospital of Shantou University Medical College.

The study population was a random cluster sample from company staff or community subjects in the Jinping district of Shantou (Guangdong, China) who were undergoing an annual physical examination in the hospital healthcare center. From 4,020 possible subjects, we recruited 596 with mild to moderate essential hypertension (sitting diastolic blood pressure 90–110 mmHg and/or sitting systolic blood pressure 140–180 mmHg from 3 consecutive measurements [19]). Only subjects who had regular sexual intercourse with marital partners were enrolled. People were excluded if they had ischemic heart disease, diabetes, chronic obstructive pulmonary disease, obstructive sleep apnea syndrome, cerebrovascular disease or other diseases that would interfere with sexual function or who used sex-enhancing medication. People using pacemakers and electrocardiography (ECG)-diagnosed atrial fibrillation/flutter were excluded. Finally, 502 subjects with hypertension were enrolled.

Subjects were given a detailed information sheet on the study procedure and were asked to give their signed informed consent to be in the study. The study was approved by the ethics committee of the First Affiliated Hospital of Shantou University Medical College. Interviews were conducted face to face on an individual basis by a trained researcher who used a structured questionnaire. The collection of all data took approximately 20 min. Patient data were strictly anonymous.

From the same population, we matched subjects with hypertension by age and sex with eligible subjects with normotension as controls and included 173 controls.

### Sociodemographics

The investigator collected data on age, sex, occupational status, education and income for both normotensive and hypertensive subjects. Daily alcohol consumption, cigarette smoking and tea drinking were classified as never, moderate or heavy. We defined moderate smoking as fewer than 5 cigarettes/day and heavy smoking as 20 or more cigarettes/day [20]. Moderate and heavy alcohol consumption were defined as total intake of alcohol, beer or wine < 20 g/day and > 20 g/day, respectively [21].

### Sexual activity questionnaire

Subjects completed a self-administered questionnaire about sexual function and activity, as well as satisfaction, that was designed to fit the local culture. It was modified and validated in preliminary tests. It consisted of 9 items: “How frequently do you usually have sexual activity with your partner in a month?”, “How frequently do you experience orgasm in a month?” “How many times do you consider your sexual activity satisfactory?” “How long is your usual sexual encounter in minutes?” “Which is the usual coital position adopted: man on top, woman on top or other?”, “How do you feel during the sexual activity: comfortable, having chest pain, headache, discomfort?” Male subjects were additionally asked “How long can your erection be maintained in minutes?”

### Physical checkup

Height and weight were measured and the body mass index (BMI) was calculated as weight/height^2^ (kg/m^2^). Blood pressure was measured with the patient in a seated position after a 5-min rest on 3 separate visits. Patients underwent standard 12-lead ECG with use of a Nihon Kohden ECG-9130P-type electrocardiograph.

### Statistical analysis

Chi-square test was used to compare differences in categorical variables. Odds ratios and 95% confidence intervals (95% CI) were calculated to assess the likelihood of characteristics of sexual activity and life factors for hypertensive men and women. Stepwise multinomial logistic regression analyses used to evaluate potential risk factors of sexual domains. The independent variables included in the multivariate regression models were sociodemographic data and daily life habits. A *p* < 0.05 was considered statistically significant. The power of tests of sexual frequency between each subgroup was > 99% by NCSS-PASS (2005 version). Statistical analyses involved use of SPSS 15.0 (SPSS Inc., Chicago, IL).

## Results

### Demographic characters and prevalence of sexual activity

Data were available for 675 subjects who reported having regular sexuality, including 502 with hypertension (225 males, mean age 48.79 ± 7.39 years [range 31-65 years]; 277 females, mean age 48.26 ± 6.93 years [range 31-63 years]) and 173 with normotension (82 males, mean age 45.69 ± 6.58 years; 91 females, mean age 46.14 ± 7.03 years (range 32-62 years) (Table 1). All women were less educated than men and were more frequently retired or unemployed than men. The sexes differed in cigarette smoking and alcohol use. Nearly half the enrolled subjects were heavy tea drinkers. Even though the blood pressure of patients with hypertension was mild to moderate, more than half did not use anti-hypertensive medicine.

**Table 1:** Demographic and clinical characteristics of enrolled subjects

	**Hypertensive group**		**Normotensive group**	
**Demographic characteristics**	**Men (n = 225)**	**Women (n = 277)**	**Men (n = 82)**	**Women (n = 91)**
**Age, mean±SD**	48.79 ± 7.39	48.26 ± 6.93	45.69 ± 6.58	46.49 ± 7.26
**Blood pressure, mean±SD**	SBP:149.80 ± 11.13 DBP:97.74 ± 8.43	151.87 ± 11.13 95.76 ± 8.04	SBP:121.15 ± 9.70 DBP:78.90 ± 8.67	117.10 ± 11.96 73.85 ± 9.89
**Educational level**				
Primary school	34 (15.1)	139 (50.2)	7 (8.5)	8 (8.8)
High school	149 (66.2)	123 (44.4)	29 (35.3)	53 (58.2)
University	42 (18.7)	15 (5.4)	46 (56.2)	30 (33.0)
**Occupational status**				
Retired or unemployed	42 (18.7)	184 (66.4)	36 (43.9)	34 (37.4)
Professional	147 (65.3)	59 (21.3)	27 (32.9)	40 (43.9)
Physical labor	36 (16.0)	34 (12.3)	19 (23.2)	17 (18.7)
**Income (RMB)**				
≤1000	37 (16.4)	194 (70.0)	8 (9.7)	47 (51.6)
1001 ~ 3000	129 (57.3)	74 (26.7)	62 (75.6)	38 (41.8)
≥3000	59 (26.2)	9 (3.2)	12 (14.6)	6 (6.6)
**Cigarette smoking**				
Never	85 (37.8)	271 (97.8)	34 (41.5)	86 (94.5)
Moderate	28 (12.4)	6 (2.2)	21 (25.6)	3 (3.2)
Heavy	112 (49.8)	0	27 (32.9)	2 (2.3)
**Alcohol consumption**				
Never	106 (47.1)	262 (94.6)	31 (37.8)	54 (59.3)
Moderate	80 (35.6)	14 (5.1)	29 (35.4)	25 (27.5)
Heavy	39 (17.3)	1 (0.4)	22 (26.8)	12 (13.2)
**Tea drinking**				
Never	16 (7.1)	48 (17.3)	16 (19.5)	16 (17.6)
Moderate	53 (23.6)	95 (34.3)	24 (29.3)	34 (37.4)
Heavy	156 (69.3)	134 (48.4)	42 (51.2)	41 (45.0)
**Medication use**				
ACEI/ARB	32 (14.2)	23 (8.3)	−	−
β blockers	8 (3.6)	9 (3.2)	−	−
CCB	28 (12.4)	37 (13.4)	−	−
Diuretics or others	14 (6.2)	17 (6.1)	−	−
No medication	143 (63.6)	191 (69)	−	−

Data are number (%) unless indicated.

SBP, systolic blood pressure; DBP, diastolic blood pressure; ACE1/ARB, angiotensin-converting enzym 1/angiotensin receptor blocker; CCB, calcium channel blocker.

Hypertensive and normotensive subjects differed in frequency of orgasm and sexual activity, as well as duration of sexual activity (*p* < 0.01; Table 2). Of the 225 males with hypertension, 57 (25.3%) reported sexual intercourse less than once per month (low frequency), 77 (34.2%) more than once per week (high frequency), with the remaining reporting intermediate activity (medium frequency). Of women with hypertension, 102 (36.8%) reported low-frequency sexual activity and 52 (20.6%) high-frequency sexual activity. Only 15 men (6.7%) experienced no orgasm during sexual activity, which coincided with the 6.7% who reported no sexual satisfaction. However, 172 women (62.1%) reported no orgasms, but only 80 (28.9%) considered their sexual life unsatisfactory. About half of the subjects, whether with hypertension or normotension, reported sexual intercourse lasting 6 to 20 min each time, and 7.5% with hypertension and 30.1% with normotension reported the intercourse lasting < 5 min. Man on top appeared to be the favored coital position, with more than 75.5% reporting this position. In total 422 subjects with hypertension (84.1%) and 165 with normotension (95.4%) reported no discomfort during the intercourse.

**Table 2:** Sexual activity for subjects with hypertension and normotension and men and women with hypertension

	Hypertension (n = 502)	Normotension (n = 173)	χ ^2^	Men with hypertension (n = 225)	Women with hypertension (n = 277)	χ ^2^
Frequency of sexual activity (times/month)			4.22			13.97^**^
Low (≤1)	159 (31.7)	46 (26.6)		57 (25.3)	102 (36.8)	
Medium (2–3)	209 (41.6)	67 (38.7)		91 (40.4)	118 (42.6)	
High (≥4)	134 (26.7)	60 (34.7)		77 (34.3)	57 (20.6)	
Frequency of orgasm (times/month)			10.69^**^			179.27^**^
Low (≤1)	187 (37.3)	81 (46.8)		15 (6.70)	172 (62.1)	
Medium (2–3)	225 (44.8)	53 (30.6)		134 (59.6)	91 (32.9)	
High (≥4)	90 (17.9)	39 (22.5)		76 (33.7)	14 (5.0)	
Frequency of sexual satisfaction (times/month)			31.67^**^			54.91^**^
Low (≤1)	95 (18.9)	67 (38.7)		15 (6.70)	80 (28.9)	
Medium (2–3)	297 (59.2)	66 (38.2)		136 (60.4)	161 (58.1)	
High (≥4)	110 (21.9)	40 (23.1)		74 (32.9)	36 (13.0)	
Duration of sexual activity (min/time)			56.35^**^			25.17^**^
≤5	164 (32.7)	35 (20.2)		55 (24.4)	109 (39.6)	
6–20	299 (59.6)	86 (49.7)		140 (62.2)	159 (57.4)	
≥20	39 (7.8)	52 (30.1)		30 (13.4)	9 (3.0)	
Coital position			1.11			15.64^**^
Man on top	379 (75.5)	132 (76.3)		151 (67.1)	228 (82.3)	
Women on top	83 (16.5)	24 (13.8)		49 (21.8)	34 (12.3)	
Other position	40 (8.0)	17 (9.9)		25 (11.1)	15 (5.4)	
Sexual feeling						3.39
Comfortable	422 (84.1)	165 (95.4)	14.21	197 (87.6)	198 (71.5)	
Uncomfortable	79 (15.9)	8 (4.6)		28 (12.4)	79 (28.5)	

Data are number (%); ^**^P < 0.01.

### Factors associated with sexual activity in hypertensive subjects

For males with hypertension, low frequency of sexual activity and orgasms and sexual satisfaction were associated with unemployed or retired employment status than physical labor (OR 0.28 [95% CI 0.12–0.69], 0.32 [0.12–0.86], 0.33 [0.19–0.88], respectively; *p* < 0.05). As well, longer duration of sexual activity was associated with never consuming alcohol than heavy drinking (4.49 [1.28–6.41]) (Table 3). For women with hypertension, low frequency and duration of sexual activity and sexual satisfaction were associated with never drinking tea than heavy tea drinking (0.42 [0.18–0.96], 0.49 [0.24–0.98], 0.29 [0.14–0.64], respectively; *p* < 0.05). Medication use and ECG results were not associated with sexual activity for hypertensive subjects.

**Table 3:** Estimated odds ratios (OR) and 95% confidence intervals (CIs) for association of demographic characteristics and sexual activity for subjects with hypertension

	**Frequency**		**Orgasm**		**Satisfaction**		**Duration**	
	Men	Women	Men	Women	Men	Women	Men	Women
Education								
Primary school	1.15 (0.42,3.15)	0.51 (0.17, 1.52)	1.16 (0.38,3.51)	0.36 (0.11,1.12)	0.69 (0.23,2.12)	0.41 (0.13, 1.32)	0.14 (0.04,0.43)*	0.61 (0.17,2.15)
High school	0.99 (0.51,1.94)	0.86 (0.31, 2.43)	1.29 (0.62,2.68)	0.62 (0.21,1.82)	1.07 (0.52,2.23)	0.76 (0.25, 2.3)	0.31 (0.14,0.66)*	1.41 (0.42,4.74)
University	1	1	1	1	1	1	1	1
Income status								
≤1000	0.55 (0.19,1.56)	1.75 (0.39,7.84)	0.49 (0.16,1.56)	0.35 (0.07,1.68)	0.63 (0.20,1.96)	0.65 (0.13, 3.25)	0.51 (0.16,1.59)	1.17 (0.21, 6.66)
1001 ~ 3000	0.76 (0.41,1.39)	1.74 (0.43,7.09)	0.63 (0.33,1.22)	0.79 (0.18,3.39)	0.71 (0.37,1.37)	0.97 (0.22,4.39)	0.87 (0.45,1.71)	1.73 (0.33,9.03)
≥3000	1	1	1	1	1	1	1	1
Occupation								
**Unemployed or Retired**	0.28 (0.12,0.69)*	0.29 (0.14, 0.62)	0.32 (0.12,0.86)*	1.06 (0.43,2.61)	0.33 (0.19,0.88)*	0.33 (0.15, 0.74)*	1.31 (0.51,3.42)	1.74 (0.25,1.33)
Professional	0.96 (0.44,2.14)	0.65 (0.24,1.72)	0.88 (0.37,2.09)	0.85 (0.28,2.57)	0.78 (0.33,1.84)	0.39 (0.14, 1.14)*	1.52 (0.64,3.62)	1.15 (0.37,3.57)
Physical labor	1	1	1	1		1	1	1
Cigarette smoking								
Never	0.71 (0.39,1.27)	2.06 (0.25,4.62)	0.63 (0.33,1.19)	6.42 (5.10,8.16)	0.64 (0.34,1.21)	1.47 (0.02,8.91)	1.05 (0.55,1.97)	7.61 (6.35,9.21)
Moderate	0.83 (0.37,1.85)	2.45 (0.43,5.52)	0.95 (0.39,2.24)	5.69 (4.22,7.34)	1.19 (0.51,2.82)	1.29 (0.16,10.5)	0.85 (0.36,2.03)	8.41 (7.22,10.14)
Heavy	1	1	1	1	1		1	1
Alcohol consumption								
Never	1.24 (0.61,2.58)	0.15 (0.14, 0.17)	1.49 (0.67,3.32)	0.45 (0.01, 27.8)	1.61 (0.72,3.59)	0.15 (0.14,0.18)	4.49 (1.28,6.41)*	0.53 (0.03, 83.7)
Moderate	1.45 (0.68,3.03)	0.17 (0.15,0.19)	2.08 (0.92,4.71)	1.05 (0.01,70.1)	1.89 (0.84,4.30)	0.14 (0.13,0.17)	2.02 (0.89,4.55)	1.02 (0.05, 68.3)
Heavy	1	1	1	1	1	1	1	1
Tea drinking								
**Never**	1.37 (0.51,3.71)	0.62 (0.31, 1.17)	0.72 (0.24,2.14)	0.42 (0.18, 0.96)*	1.08 (0.37,3.14)	0.49 (0.24,0.98)*	0.93 (0.31,2.73)	0.29 (0.14, 0.64)*
Moderate	1.13 (0.61,2.09)	0.77 (0.46, 1.68)	0.95 (0.49,1.85)	0.67 (0.38,1.19)	0.81 (0.41,1.59)	0.76 (0.44,1.32)	0.87 (0.44,1.71)	0.73 (0.41,1.29)
Heavy	1		1	1	1	1	1	1
Medication								
Yes	1.13 (0.66,1.93)	0.84 (0.51, 1.38)	0.79 (0.45,1.43)	0.91 (0.53,1.58)	1.07 (0.59,1.92)	0.84 (0.49,1.41)	1.18 (0.65,2.12)	0.72 (0.41,1.26)
No	1		1	1	1	1	1	1
ECG result								
Normal	1.61 (0.94,2.76)	0.96 (0.59, 1.56)	1.63 (0.91,2.94)	0.69 (0.41,1.19)	1.50 (0.83,2.71)	0.91 (0.56, 1.52)	1.58(0.87,2.86)	0.64 (0.37,0.91)
Abnormal	1	1	1	1	1	1	1	1

^*^P < 0.05.

## Discussion

Hypertension is an important factor contributing to SD. Although the number of people with hypertension is increasing in China, research into sexual life, with implications for quality of life, is limited. We compared sexual activity and the influence of daily behaviors and sexual factors of hypertensive and normotensive males and females in south China and found that lifestyle factors dictated SD in this area. Hypertensive and normotensive subjects differed in frequency of orgasms and sexual satisfaction, as well as duration of sexual activity. For hypertensive men, low frequency of sexual activity and orgasms and sexual satisfaction were associated with unemployed or retired status than physical labor work, and long sexual duration was associated with never drinking alcohol than heavy drinking. For hypertensive women, low frequency and duration of sexual activity and low satisfaction were associated with never drinking tea than heavy tea drinking.

Hypertension, especially mild to moderate, is usually considered an asymptomatic condition [22]. Reports of quality of life with hypertension by the Medical Outcomes Survey SF-36 are abundant in western countries. In China, quality of life was greater for people with normotension than hypertension and greater for patients receiving treatment and with controlled hypertension than for those with poorly controlled hypertension [23]. However, these studies did not focus on sexual activity.

Our results were similar to those of the Caerphilly study of frequency of sexual intercourse for 914 males in the United Kingdom [24]: 197 (21.5%) had sexual intercourse less than once per month, 231 (25.3%) twice or more per week, and the remaining 486 (53.2%) reported intermediate frequency. For our males, the frequency of orgasm was comparable to the reported satisfaction, which suggests that the assessment of sexual satisfaction was mainly based on orgasm experience. Almost two-thirds of females experienced no orgasm, but only about one-third were unsatisfied with sexual encounters, which is consistent with reports of middle-aged and older women in the United States [25]. Indeed, female satisfaction in sexual intercourse in both western and Asian societies is related more to the emotional than physical experience [26].

The decreased number of orgasms we found for hypertensive females may be associated with most females being in peri-or postmenopause status. As well, the low sexual activity for females might be the result of declining estrogen and testosterone levels affecting sexual desire [25]. In a cohort of older postmenopausal women (mean age 68 years) with osteoporosis, at baseline, only 46% reported some sexual activity; the most-reported problem was difficulty in achieving orgasm [27]. Sex hormones are essential for sex-specific behaviors [28], and levels of oestrogens and androgens do account for gender differences in sexuality [29].

We found that sexual activity decreased with age. Because most unemployed or retired men were older subjects, their sexual frequency, orgasm and therefore satisfaction were lower than for younger subjects. We found less tea drinking related to low number of orgasms, satisfaction and sexual duration for hypertensive women. Clinical studies of men suggest that tea drinking may inhibit the proliferation of smooth muscle cells and overall improvement in endothelial function, which may play a role in erectile function. An epidemiological survey found that long-term green tea consumption changed androgen levels in women. However, the precise nature of the association of tea drinking and sexuality needs further study.

In an American study [30], women with high levels of education and income reported a high frequency of sexual activity. As well, those with at least monthly sexual frequency were more likely to have never smoked or currently drink alcohol and had better health status. Results from our study may differ from the previous results because Chinese women tend to drink tea rather than alcohol and to not smoke. Alcohol drinking is a risk factor for hypertension. Recent research of Chinese men found that compared with non-alcohol drinkers, alcohol drinkers who consumed 3 or more drinks a week (1 drink equals 12 g of alcohol) were more likely to report erectile dysfunction as defined by having both sexual dissatisfaction and erectile difficulty (OR = 2.27) [31]. In our hypertensive subjects, heavy alcohol consumption was associated with short duration of sexual activity.

## Conclusions

The prevalence of sexual activity differs for men and women with hypertension in China. Lifestyle factors, including educational level, income status and tea drinking may be associated with sexual activity for women and occupational status and alcohol consumption for men. However, further studies are needed with larger samples and investigating serum hormone levels to validate these results.

### Limitations

The main limitation of this study was the self-reporting methodology. Self-reported sexual frequency may be higher than reported because patients may give a socially desirable answer about their sexual function. The items in the self-developed questionnaire may not be sufficient, but enrolled subjects might not be cooperative enough to complete a long questionnaire in the hospital. In addition, several other factors not measured, including side effects of anti-hypertensive medication, may influence sexual activity among people with hypertension, although two-thirds of our subjects with hypertension took no medications. Finally, the sample size of the normotensive group was relatively small as compared with the hypertensive group because most people with normotension were unwilling to participate in the study. Persuading healthy people to undergo a medical checkup and provide answers to sensitive issues, even with payment is difficult.

## Misc

Xiaojun Chen and Qingying Zhang contributed equally to this research work

## Competing interest

The authors declare no conflict of interest.

## Authors’ contributions

XC performed the survey and drafted the manuscript. QZ supervised data collection and helped draft the manuscript. XT designed the study and helped interpret the results and modify the manuscript. All authors read and approved the final manuscript.

## Pre-publication history

The pre-publication history for this paper can be accessed here:

http://www.biomedcentral.com/1471-2458/12/364/prepub
